# FUS is lost from nuclei and gained in neurites of motor neurons in a human stem cell model of VCP-related ALS

**DOI:** 10.1093/brain/awaa339

**Published:** 2020-11-30

**Authors:** Jasmine Harley, Cathleen Hagemann, Andrea Serio, Rickie Patani

**Affiliations:** 1 The Francis Crick Institute, London NW1 1AT, UK; 2 Department of Neuromuscular Diseases, UCL Queen Square Institute of Neurology, Queen Square, London, UK; 3 Centre for Craniofacial and Regenerative Biology, King’s College London, London, UK

##  

Amyotrophic lateral sclerosis (ALS) is a rapidly progressive and uniformly fatal neurodegenerative disease characterized by the loss of motor neurons. Precise underlying disease mechanisms remain incompletely resolved but key hallmarks of the disease include the mislocalization of ubiquitously expressed RNA binding proteins (RBPs) from the nucleus to the cytoplasm. Until recently mislocalization of the RBP fused in sarcoma (FUS) in ALS was predominantly a recognized feature of only FUS mutation-related ALS (Vance *et al.*, 2009). However, in our recent *Brain* paper, we reported widespread mislocalization of wild-type FUS in familial and sporadic forms of ALS (Tyzack *et al.*, 2019). Specifically, we examined a form of ALS caused by mutations in the valosin containing protein (*VCP*) gene in both human induced pluripotent stem cell (hiPSC) cultures and a mouse transgenic model. In the same study, we also reported FUS mislocalization in sporadic ALS post-mortem tissue, confirming that it is a more widespread hallmark than previously recognized (Tyzack *et al.*, 2019). However, in the hiPSC model we only examined the nuclear-to-cytoplasmic ratio of FUS within neural precursors before confirming our findings in motor neurons from mouse transgenic and human post-mortem tissue sections. Therefore, our study left unresolved whether FUS mislocalization (i) could be recapitulated in hiPSC-derived terminally differentiated motor neurons; and (ii) whether cytoplasmic FUS can be detected within the neuronal processes themselves or if it is restricted to the soma. This is challenging to study in tissue sections as the arborization of processes is largely lost during sectioning. To address this issue, we used our established directed differentiation protocol to generate highly enriched spinal motor neurons from hiPSCs, which has previously been extensively validated for cellular identity and functionality (Hall *et al.*, 2017), together with its ability to faithfully recapitulate ALS phenotypes (Luisier *et al.*, 2018; Tyzack *et al.*, 2019; Smethurst *et al.*, 2020).

We investigated the subcellular localization of FUS in hiPSC-derived terminally differentiated motor neurons using immunolabelling and fluorescence microscopy. Semi-automated image analysis was undertaken using machine learning-based pixel classification (Ilastik; Berg *et al.*, 2019) for nuclear detection and an automated image analysis pipeline (Cellprofiler; Carpenter *et al.*, 2006) for masking neurites (Fig. 1A). Using this automated and unbiased analysis method we were able to detect a significant alteration in the nuclear-to-cytoplasmic ratio of FUS within terminally differentiated hiPSC-derived VCP-mutant motor neurons compared to their control counterparts (Fig. 1B). We demonstrate that the increase in extranuclear FUS is significant within neuronal processes of VCP-mutant motor neurons (Fig. 1C and D). Additionally, we detect a decrease in nuclear FUS (Fig. 1E).

**Figure 1:**
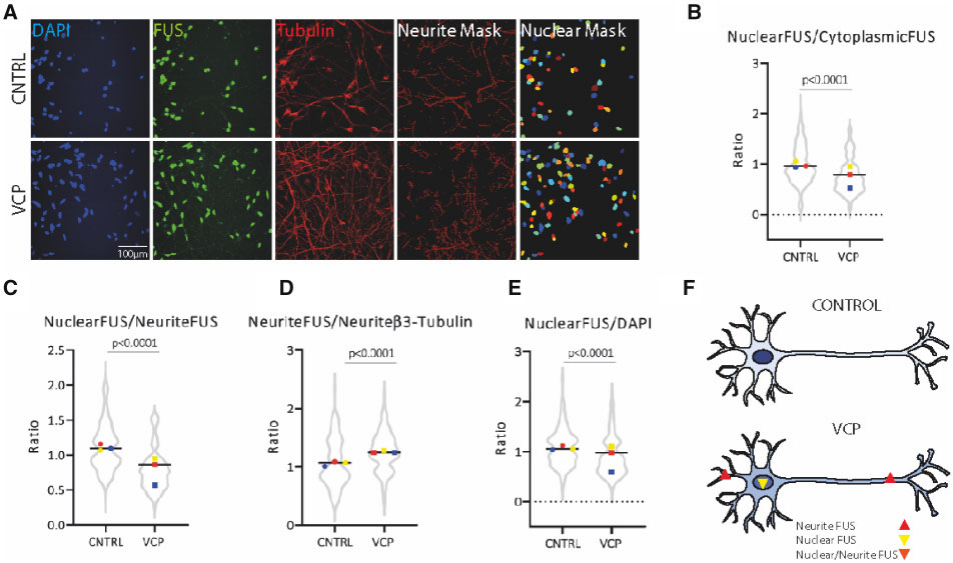
**FUS is mislocalized in hiPSC-derived VCP-mutant motor neurons.** (**A**) Subcellular localization of FUS in hiPSC-derived motor neurons with accompanying semi-automated image analysis segmentation masks for neurites, based on β-III-tubulin immunolabelling, and nuclei based on DAPI staining. (**B**) Quantification of FUS immunolabelling in the nucleus and the cytoplasm reveal that VCP-mutant motor neurons exhibit a decrease in FUS nuclear-to-cytoplasmic ratio. (**C**) Quantification of FUS in the nucleus and neurites reveal that VCP-mutant motor neurons exhibit a decrease in nuclear-to-neurite ratio of FUS. (**D**) FUS quantification in the neurites normalized to β-III-tubulin shows that VCP-mutant motor neurons exhibit a gain of FUS in the neurites. (**E**) Normalization of nuclear FUS to DAPI shows a nuclear loss of FUS in VCP-mutant motor neurons. Data are presented as ratio over control values for each experimental repeat, with each experimental repeat plotted and individual values displayed within the violin plot. Three independent control lines and four VCP-mutant lines (two clones from each of two patients) from three independent experiments were used to generate these data. *P*-values were calculated from an unpaired Mann-Whitney test. Intensity values were calculated using the integrated intensity divided by the area and as a background subtraction we used the lower quartile intensity per image and object. (**F**) Schematic diagram summarizing the mislocalization of FUS protein in VCP-mutant motor neurons.

Here we provide evidence of a decrease in the nuclear-to-cytoplasmic ratio in VCP-mutant motor neurons, which reinforces the notion that wild-type FUS nuclear-to-cytoplasmic mislocalization—rather than its overt and pronounced aggregation—is a more widespread feature of of ALS beyond just cases caused by FUS mutations (Tyzack *et al.*, 2019). The nuclear loss of FUS protein may impair its fundamental role in pre-mRNA splicing, which has been linked to neurodegeneration (Kino *et al.*, 2011; Ishigaki and Sobue, 2018). In the present study, detailed image analysis of the motor neuron cytoplasmic area also allowed us to extend our previous findings by demonstrating that wild-type FUS is significantly increased within neuronal processes in VCP-mutant motor neurons (Fig. 1A–F). Importantly, because of the compartmentalization of the axon and dendrites from the soma, simple diffusion is unlikely to explain this finding and an increase in active axonal transport is a more likely explanation. It is noteworthy that mutant FUS within axons was recently found to perturb local protein translation and to be driving pathogenesis independently of a nuclear loss of its function (López-Erauskin *et al.*, 2018). The precise molecular mechanisms and consequences of FUS mislocalization (nuclear loss of function and/or cytoplasmic gain of function) have yet to be fully resolved; however, we demonstrate that hiPSC-based models can be used to more precisely dissect the sequence of molecular events underlying this process.

In summary, we report that hiPSC-derived motor neurons carrying the VCP mutation exhibit a nuclear-to-cytoplasmic mislocalization of wild-type FUS protein, which extends to the neuronal processes (Fig. 1F). These findings raise the prospect of targeting the cytoplasmic FUS as a putative therapeutic strategy in forms of ALS beyond just those caused by FUS mutations. Our study further highlights the utility of hiPSC-derived motor neurons for studying ALS pathomechanisms and for drug discovery through compound screening in a clinically relevant and experimentally tractable model.

### Data availability

The data that support the findings of this study are available from the corresponding authors, upon reasonable request.

## Funding

This work was supported by the Francis Crick Institute which receives its core funding from Cancer Research UK (FC010110), the UK Medical Research Council (FC010110), and the Wellcome Trust (FC010110). R.P. holds an MRC Senior Clinical Fellowship [MR/S006591/1]. A.S acknowledges the support of the Wellcome Trust [213949/Z/18/Z].

## Competing interests

The authors report no competing interests.
